# The paradoxical need for human intervention in the conservation of natural environments in Venice lagoon

**DOI:** 10.1038/s41598-023-33754-3

**Published:** 2023-04-26

**Authors:** Alice Stocco, Fabio Pranovi

**Affiliations:** Department of Environmental Sciences, Statistics and Informatics, Ca’ Foscari University of Venice, 30174 Venice Mestre, Italy

**Keywords:** Ecosystem services, Sustainability, Wetlands ecology

## Abstract

The Venice lagoon—the largest Mediterranean coastal lagoon—is characterized by the presence at its edges of 31 “valli da pesca”, types of artificial ecosystems that mime the ecological processes of a transitional aquatic ecosystem. Constituted by a series of regulated lakes bounded by artificial embankments, the valli da pesca were established centuries ago to maximize provisioning Ecosystem Services (ESs), such as fishing and hunting. As time passed, the valli da pesca underwent an intentional isolation process leading to private management. Nonetheless, the valli da pesca are still exchanging energy and matter with the “open’ lagoon and today represent an essential element within the context of lagoon conservation. This study aimed to analyze the possible effects of artificial management on both ESs supply and landscape arrangements by assessing 9 ESs (climate regulation, water purification, lifecycle support, aquaculture, waterfowl hunting, wild food, tourism, information for cognitive development, and birdwatching), along with eight landscape indicators. Obtained results suggested that the valli da pesca are today ruled under five different management strategies, according to the maximized ES. Management conditions influence the landscape pattern and achieve a series of “side effects” on the other ESs. The comparison between the managed and abandoned valli da pesca highlights the importance of anthropogenic interventions for conserving these ecosystems, as the abandoned valli da pesca show a loss of ecological gradients, landscape heterogeneity, and provisioning ESs. Nevertheless, the persistence of intrinsic geographical and morphological characteristics still prevails regardless of intentional landscape molding. The result is that the provisioning ESs capacity per unit area is higher in the abandoned valli da pesca than in the open lagoon, emphasizing the importance of these confined areas of the lagoon ecosystem. Considering the spatial distribution of multiple ESs, the provisioning ESs flow that does not occur in the abandoned valli da pesca seems to be replaced by the flow of cultural ESs. Thus, the ESs spatial pattern highlights a balancing effect between different ESs categories. The results are discussed considering the trade-offs generated by private land conservation, anthropogenic interventions, and their relevance for the ecosystem-based management of Venice lagoon.

## Introduction

Traditionally recognized as highly productive ecosystems^1–3^, coastal lagoons and deltas have always experienced the effects of multiple interactions between humans and the environment^4–12^. Among the coastal lagoons of the Mediterranean Sea, the Venice lagoon represents a good example of a complex social-ecological system subjected to a long co-evolution process of such interactions. The peculiar lagoon environment, synchronized with the daily tidal regime and seasonal biological cycles, played a key role in shaping human settlements, which occurred in different areas depending on the activity to be delivered. While residential safety was sought on the islands through ground consolidation techniques^13^, other modifications to the landscape were devoted to facilitating food provisioning^14–16^.

Since remote times^16,17^ in Italy as well as elsewhere^17–22^, people have observed fish and waterbird migrations and identified the areas at the interface between land and water as the places where these species naturally concentrate^15,23^. These areas, where land, rivers, and lagoon water meet, have been seen as forefronts where fishing and hunting can be carried out more easily and effectively^24–27^. Therefore, people have begun to exploit them by adopting temporary closures and entrapment systems^19,28–30^.

**Figure 1:**
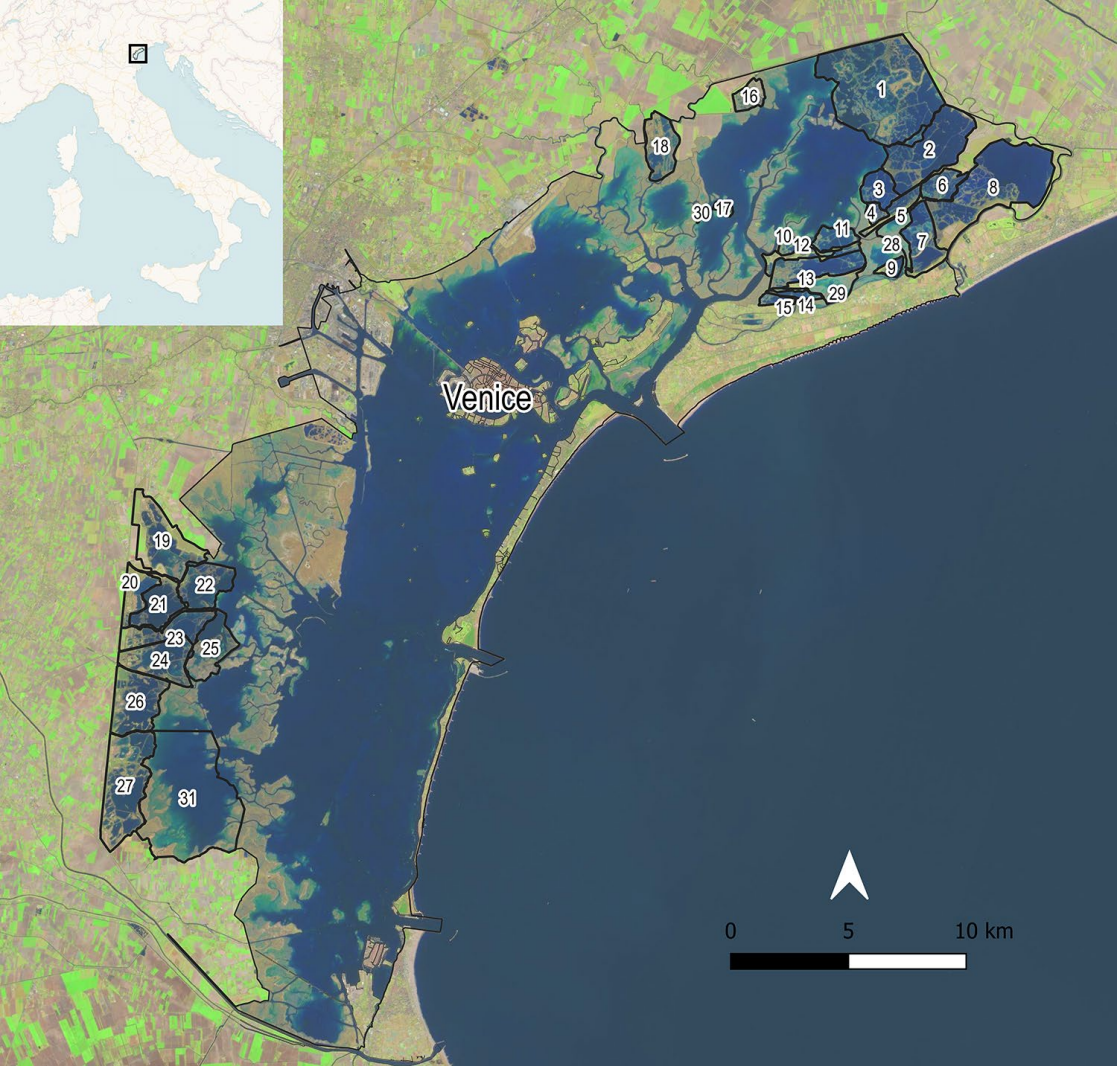
The valli da pesca of the Venice lagoon. 1 = Valle Dogà, 2 = Valle Grassabò, 3 = Vallesina, 4 = Valle Fosse, 5 = Valle Lio Maggiore, 6 = Valle Bianca, 7 = Valle Dragojesolo, 8 = Valle Cavallino, 9 = Valle Falconera, 10 = Valle Liona, 11 = Valle Olivara, 12 = Valli Saline-Manciane-Sparasera, 13 = Valle Paleazza, 14 = Valle Sacchettina, 15 = Valle Sacchetta, 16 = Valle Ca' Zane, 17 = Santa Cristina island, 18 = Valle Perini, 19 = Valle Miana-Serraglia, 20 = Valle Averto, 21 = Valle A.M.A., 22 = Valle Contarina, 23 = Valle Cornio Alto e Cornio Basso, 24 = Valle Zappa, 25 = Valle Figheri, 26 = Valle Pierimpiè, 27 = Valle Morosina-Ghebo Storto, 28 = Valle Baseggia, 29 = Valle delle Mesole, 30 = La Cura, 31 = Valle Millecampi. Map elaborated from Sentinel-2A scene collected in 2022, March 23^rd^.

In the Venice lagoon, local ecological knowledge and empirical observations have inspired the use of nets and temporary reed fences to facilitate fish trapping in the most confined part of the lagoon ecosystem. This gave origin to the Italian “valli da pesca” ^31^, where capture-based extensive aquaculture and hunting reserves were established (Fig. 1).

Over the decades, permanent dikes have replaced the temporary boundaries and separated the valli da pesca from the main part of the Venice lagoon^31–33^, leading the valli da pesca to become artificially managed as if they were productive areas. On the one hand, such a change drove the valli da pesca to rely on men’s efforts for their functioning and maintenance, especially for water flow and fish recruitment. On the other hand, implementing artificial management in the valli da pesca allowed for conserving these areas through empirical nature-based solutions, namely saltmarshes restoration, channel dredging, fence planting, levees reinforcement with ripraps. This allows them to maintain their capability to preserve, within their reinforced boundaries, the typical elements of a transitional water ecosystem, such as saltmarshes and salinity gradients, as well as the related ecological processes. Consequently, the valli da pesca continue to represent important wintering and nesting areas^34–37^ making a noticeable contribution in terms of Ecosystem Services^38,39^ (ESs) to the entire lagoon ecosystem, even if they have been privately managed and subjected to anthropogenic interventions for a long time.

Indeed, the valli da pesca today contribute to the lagoon ESs supply, representing 38% of the total potential to provide ESs and ensure 24% of the total flow to the local community, although they cover only 17% of the lagoon surface^40^.

Considering the great importance of these confined and overlooked areas of the lagoon, we focused on analyzing the ESs supply pattern under different human management condition of the 31 valli da pesca in the Venice lagoon. Our principal aim was to verify whether and how different management conditions in the valli da pesca imply distinctive anthropogenic modifications, capable of influencing their ecosystems’ structure and so their ESs.

To do so, we assessed nine ESs belonging to the “regulating and maintenance”, “provisioning”, and “cultural” ESs categories^41^ by evaluating their *capacity* and *flow*^42^. This approach allows us to investigate whether different management strategies influence the potential of these ecosystems to deliver multiple ESs (capacity), the amount of ESs that reaches society (the flow), or both ^42,43^.

We classified the valli da pesca into five groups that indicate the management strategies in terms of ESs maximization. Indeed, the most important ES at which the management aims was identified according to the managers’ statements and validated with data regarding fish production, hunting catches, and hosted tourists and excursionists.

In addition, we described the landscape features that characterize each of the valli da pesca through landscape indicators. Multivariate statistical analyses allowed us to detect, on the one hand, whether different artificial management strategies led to significant peculiarities in landscape arrangement and, on the other hand, how such changes can have consequences on the ESs.

We hypothesized that the maximization of one category of ESs, changing both the landscape features and the processes in the valli da pesca through anthropogenic interventions, could have consequences for other ESs categories that need to be assessed to avoid imbalances or sustainability issues. Furthermore, such an assessment could provide a valuable framework to describe which anthropogenic modifications are effective in allowing the exploitation of a transitional aquatic ecosystem for provisioning or cultural ESs while avoiding affecting its regulating ESs capacity.

## Results

### Classification of the valli da pesca as managed ecosystems

The ESs assessment and interviews showed that the valli da pesca managers usually maximize only one or a few ESs. Consequently, the 31 valli da pesca can be classified into five groups according to the adopted management strategy and maximized ESs (Table 1).

**Table 1:** The five groups of valli da pesca, according to the adopted management strategy.

Group	Legend no	Names of the valli da pesca
Fish production (F)	1, 2, 13, 21	Val Dogà, Valle Grassabò, Valle Paleazza, Valle A.M.A
Multiple ESs (M)	5, 7, 8, 18, 22, 23, 25, 26	Valle Lio Maggiore, Valle Cavallino, Valle Dragojesolo, Valle Perini, Valle Contarina-Tezze, Valle Cornio, Valle Zappa, Valle Pierimpiè
Hunting activity (H)	3, 4, 6, 10, 11, 16, 19, 24, 27	Lago Vallesina di Grassabò, Valle Bianca, Valle Fosse, Valle Olivara, Valle Liona, Valle Ca’ Zane, Valle Miana-Serraglia, Valle Figheri, Valle Morosina
Recreational activities (R)	9, 12, 14–15, 17, 20	Valle Falconera, Saline-Manciane-Sparasera, Valle Sacchetta -Sacchettina, Santa Cristina, Valle Averto (WWF Oasis)
Not managed (N)	28, 29, 30, 31	Valle Baseggia, Mesole, La Cura, Valle Millecampi

Group “F” is composed of the valli da pesca that adopted the strategy to prioritize the maximization of fish production from extensive aquaculture. Group “M” is composed of the valli da pesca that are managed to exploit aquaculture and hunting, sometimes adding tourist opportunities. Group “H” identifies the valli da pesca that enhance hunting activity. The valli da pesca that no longer rely on provisioning ESs and base their management on maximizing cultural ESs represent the group “R”. Group “N” includes valli da pesca that are no longer managed.

### Ecosystem services indicators analyses

**Figure 2:**
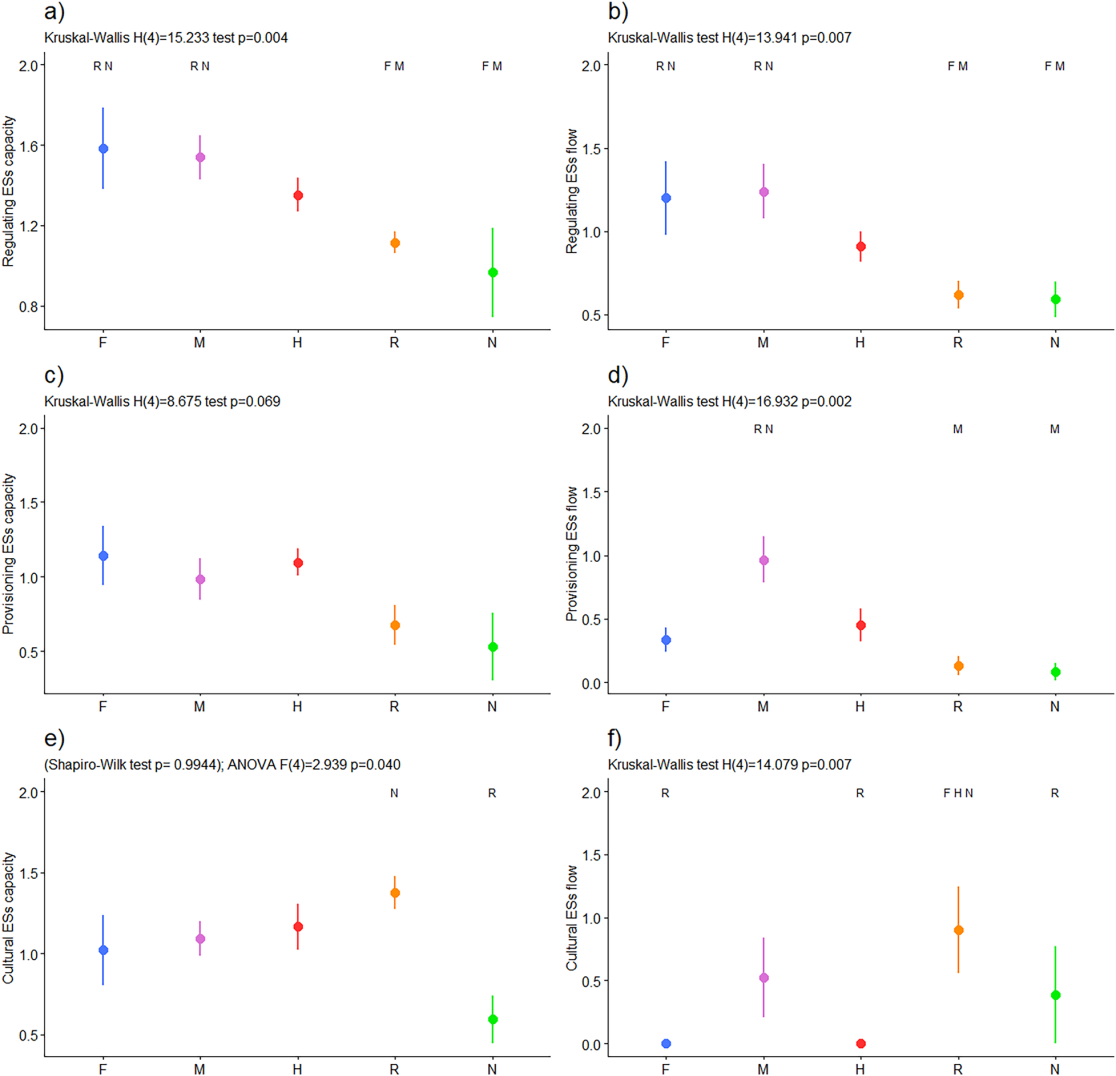
Ecosystem services aggregated indicators. The dots represent the means; the error bars show each group’s standard errors of ESs indicators. In the x-axis are reported the five groups: F = fish production; M = Multiple ESs; H = Hunting; R = Recreational; N = Not managed. The letters on the top of the plots indicate the group(s) to which the group is significantly different, at 95% confidence level. Panel (**a**) shows Regulating and Maintenance ESs capacity; (**b**) shows Regulating and Maintenance ESs flow; (**c**) shows Provisioning ESs capacity; (**d**) shows Provisioning ESs flow; (**e**) shows Cultural ESs capacity; (**f**) shows Cultural ESs flow.

Analyses of the aggregated ESs indicators show that the valli da pesca of groups F, M, and H have higher values of regulating and maintenance ESs than those of groups R and N, both for capacity and flow. Group F significantly differed from R and N in terms of capacity (*p-*value 0.007 and 0.009, respectively) and R and N flow (*p-*value 0.007 and 0.006, respectively). Group M showed a similar pattern to R and N capacity (*p-*value 0.004 and 0.006, respectively) and R and N flow (*p-*value 0.006 and 0.009, respectively), as shown in Fig. 2a,b.

The capacity indicator of provisioning ESs did not show statistically significant differences among the groups (Fig. 2c), even though groups F, M, and H showed higher values than R and N. In contrast, the flow indicator of group M had a significantly higher value than those of groups R and N (*p-*value 0.00070, 0.00074), as shown in Fig. 2d.

The cultural ESs capacity resulted in a lower value in group N when compared to the capacity of the managed valli da pesca of groups F, M, H, and R (Fig. 2e). Among the managed groups, the cultural ESs capacity reached its highest value in group R, for which the flow was consistently higher than the flow in F (*p-*value 0.004), H (*p-*value 0.001), and N (*p-*value 0.046). In particular, groups F and H had no flow, whereas limited flow was observed in group M (Fig. 2f).

**Figure 3:**
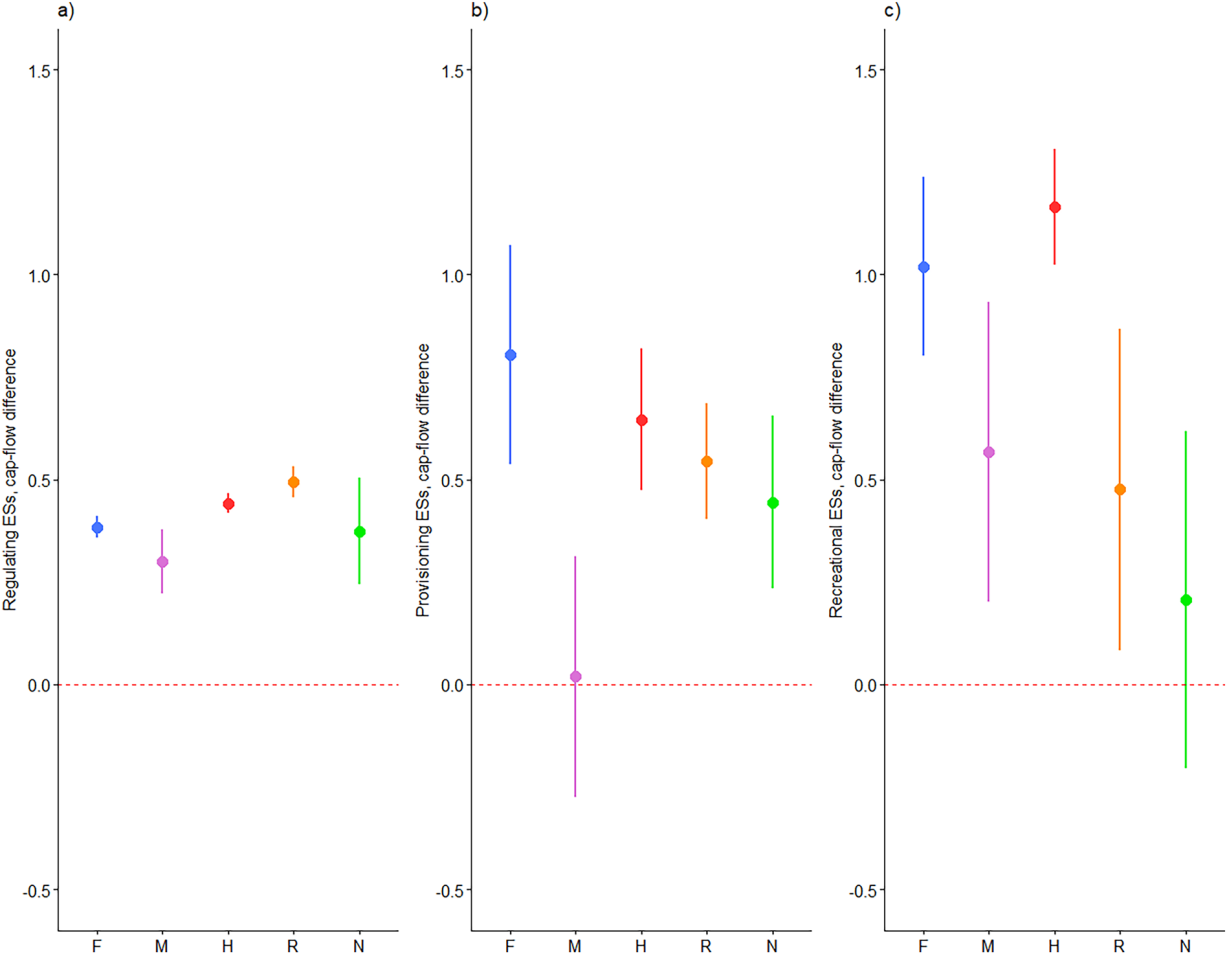
Ecosystem services capacity-flow difference. The dots represent the means; the bars represent the standard errors of the indicator for each group. In the x-axis are reported the five groups: F = fish production; M = Multiple ESs; H = Hunting; R = Recreational; N = Not managed. Panel (**a**) shows Regulating and Maintenance ESs capacity-flow difference. Panel (**b**) shows Provisioning ESs capacity-flow difference. Panel (**c**) shows Cultural ESs capacity-flow difference.

On average, all groups had a capacity-flow difference higher than zero (Fig. 3). However, group M showed a low capacity-flow difference (Fig. 3b) due to provisioning ESs flow higher than capacity in two valli da pesca. This can be noticed, as well, considering the ESs capacity and flow values in each of the areas through the stellar charts available in the Supplementary Information section (S1).

**Figure 4:**
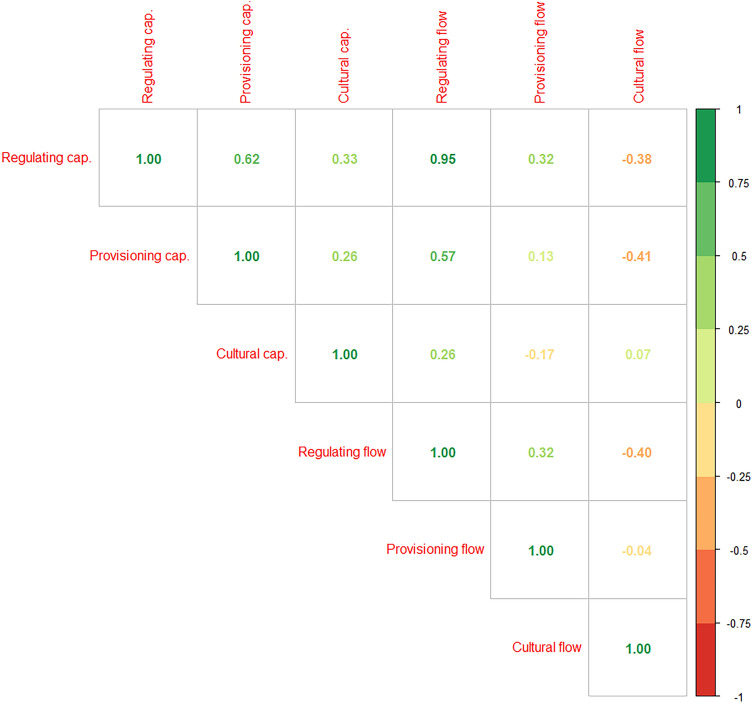
Correlogram between different ESs capacity and flow indicators. Reported numbers refer to Spearman’s **ρ** coefficient: green color highlights positive coefficient, red color highlights negative ones. Details about significance in the text.

The results of the spatially explicit ESs assessment and the ESs aggregate indicators analyses suggested the presence of interactions between different ESs categories. Groups that maximize provisioning ESs do not sustain cultural ESs, and vice versa. Regulating ESs seemed to be associated more with provisioning ESs maximization than with cultural ESs maximization. The exploratory correlation analysis among ESs aggregated indicators detected a significant positive correlation between the capacity and flow of regulating ESs (Spearman’s rho = 0.95, *p-*value = 2.2e^−16^, Fig. 4). In contrast, no linear correlation arises between provisioning ESs capacity and flow, nor between cultural ESs capacity and flow. Regarding the relationships between different categories of ESs, we detected a positive Spearman’s correlation between provisioning ESs capacity and regulating ESs capacity (**ρ** = 0.62, *p-*value = 0.0002), as well as between provisioning ESs capacity and regulating ESs flow (**ρ** = 0.57, *p-*value = 0.001).

Instead, a negative correlation arises between the capacity of provisioning ESs and the flow of cultural ESs (**ρ** = − 0.41, *p-*value = 0.024).

### Landscape characteristics analysis

**Figure 5:**
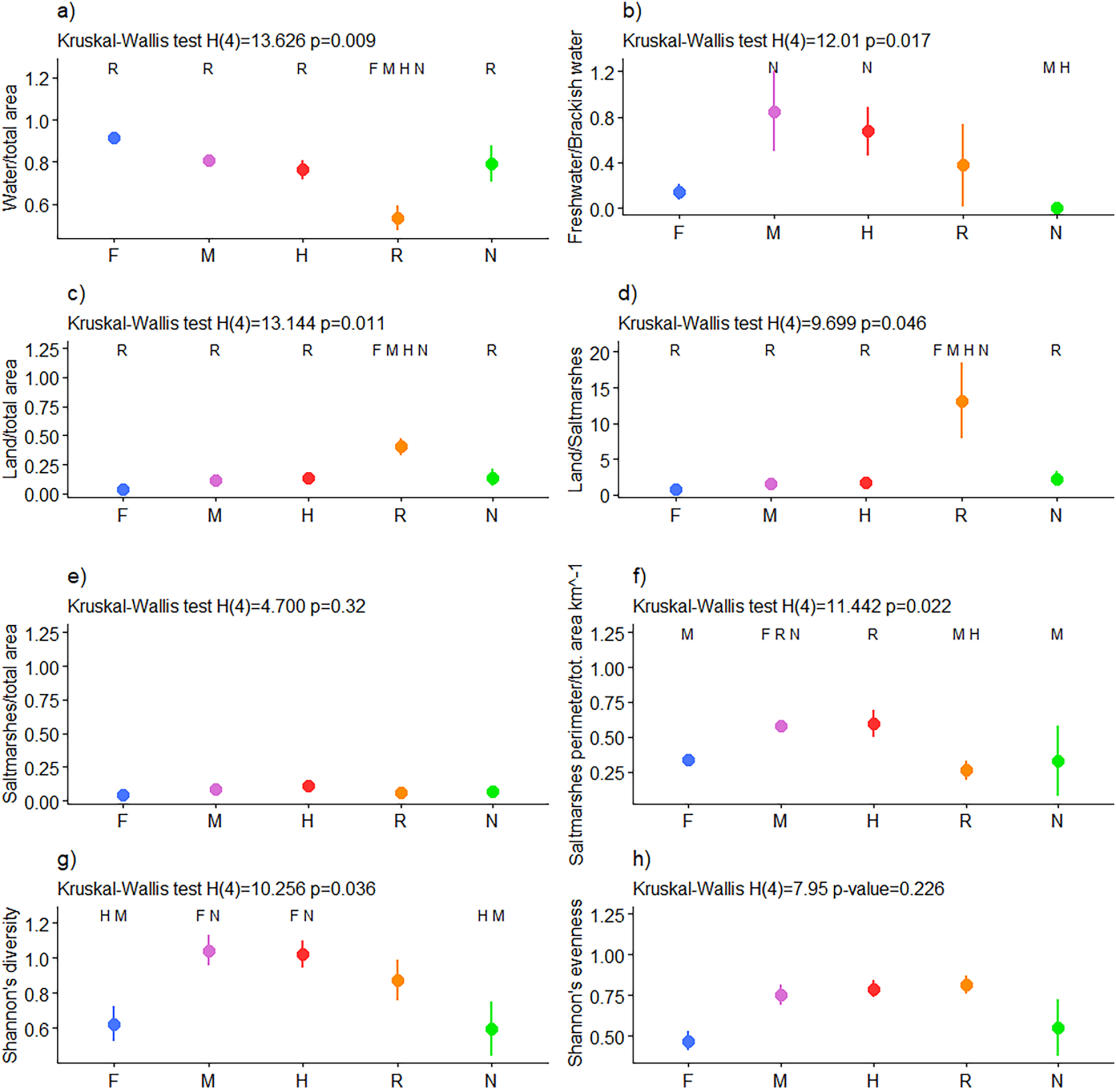
Landscape aggregated indicators. The dots represent the means; the bars represent each group’s standard errors of ESs indicators. In the x-axis are reported the five groups: F = fish production; M = Multiple ESs; H = Hunting; R = Recreational; N = Not managed. The letters on the top of the plots indicate the group(s) to which the group is significantly different, at 95% confidence level. Panel (**a**) shows ratio between water covered area and total area of the valle da pesca; (**b**) shows ratio between freshwater covered area and brackish water covered area; (**c**) shows ratio between land area and total area of the valle da pesca; (**d**) shows ratio between land area and saltmarshes area; (**e**) shows ratio between saltmarshes area and total area of the valle da pesca (km^−1^); (**f**) shows ratio between saltmarshes perimeter and saltmarshes area; (**g**) shows landscape Shannon’s diversity index; (**h**) shows landscape Shannon’s evenness index.

Seven out of eight landscape indicators showed significant differences among the groups. As shown in Fig. 5a, group F had the highest ratio between the water-covered surface and the total area; groups M and H followed along with group N; only group R had a low water area/total area ratio, which significantly differed from all the other groups (R vs. F: *p-*value 0.0002; R vs. M: *p-*value 0.017; R vs. H: *p-*value 0.031; R vs. N: *p-*value 0.032). Most of the water-covered area is represented by brackish basins that only in some groups are interspersed with freshwater lakes. As a result, a freshwater/brackish water ratio results in a significant difference between groups N and H (M vs. N: *p-*value 0.006; H vs. N: *p-*value 0.006) (Fig. 5b).

In the valli da pesca of group R, terrestrial land represented most of the surface (Fig. 5c), driving the ratio between land and saltmarshes to the highest value (Fig. 5d). A peculiar pattern can be noticed focusing on saltmarshes geometry: even if saltmarshes cover similar surfaces in all groups (Fig. 5e), their geometry varies considerably under different management strategies. As shown in Fig. 5f, the edge length of saltmarshes per unit area was higher in groups M and H.

All these features make a significant difference also in the landscape heterogeneity. Figure 5g shows that the average Shannon’s diversity index for groups M and H is considerably higher than F and N index values, and slightly higher than the value of group R (F vs. M: *p-*value = 0.018, F vs. H *p-*value = 0.023, M vs. N: *p-*value = 0.025, H vs. N: *p-*value = 0.031).

F and N also presented Shannon’s landscape evenness similar to the other groups; however, no significant differences were observed among groups in terms of evenness or richness in M and H (Fig. 5h).

Aerial images of the ground-truth landscape elements considered for calculating the landscape indicators are available in Supplementary Material (S2, Figs. S2.1–S2.5).

## Discussion

Coastal areas have always provided a plethora of ESs for human well-being. Among coastal ecosystems, coastal lagoons and transitional aquatic ecosystems have been exploited for aquaculture purposes since ancient times^18,20–22,33,44–47^.

In Italy, traditional ecological knowledge about the migratory behavior of fish that migrate from the sea to shallow brackish waters ^36,37,48–50^ has driven the development of particular enclosures to entrap juvenile fish and hold them until they reach commercial size^32,51–54^. The Venice lagoon made no exception, and today is home to the most emblematic examples of this type of extensive capture-based aquaculture, which often integrates with waterfowl hunting^20,31,33^. These systems, called valli da pesca, initially presented thin mobile barriers, but permanent dikes and boundaries were soon built to protect them. However, the decision to isolate these portions of the lagoon ecosystem implied the necessity of artificially maintaining their functional elements by ensuring the exchange of freshwater and saltwater on the landside and the lagoon side, respectively. Thus, the valli da pesca started to be managed as artificial ecosystems, where the maintenance of water fluxes, salinity gradients, and landscape elements became dependent on human work.

The valli da pesca, being artificially managed, have been excluded from most of the studies concerning the lagoon of Venice; therefore, their conditions and environmental characteristics are poorly known. Nevertheless, they represent examples of how anthropogenic modifications in an ecosystem can have several effects on both the ESs supply and landscape.

To understand the effects of anthropogenic management of ecosystems using nature-based practical interventions, we adopted a combined approach using ESs and landscape indicators.

The highlighted relationship between ESs, landscape arrangement, and anthropogenic interventions confirms that analyses of ESs are essential to frame the actual value of a managed ecosystem. Moreover, ESs assessments inform the sustainable use of services and resources, especially when managing highly modified ecosystems with a prominent role in a complex social-ecological system.

Our analyses highlighted that managing the valli da pesca allows for a higher ESs capacity than in the absence of management. The valli da pesca that are no longer managed (group N) show a provisioning ESs capacity that, despite being the lowest among the five groups, still tends to be 3.77 times higher than the capacity per unit area in the open lagoon. The main reason for the potential to provide ESs could be, apart from the persistence of morphological and geographical factors that make the confined lagoon areas attractive for fish and birds, the remnants of the structures of the valli da pesca. Such structures are estimated to be still capable of influencing the hydrodynamics of the area^55^ and probably offer some resilience both in slowing the loss of environmental heterogeneity and in preventing excessive pressures, even after the end of human work. The management of these confined areas in the lagoon, even if applied for a period and then discontinued, could thus have an interesting effect on increasing the natural capacity for provisioning ESs through anthropogenic interventions, which maintain some features that imitate the “original” lagoon processes.

A side effect of this outcome is that, in managed valli da pesca, the capacity to offer cultural ESs is high because of the maintenance of those characteristics that work as attractive features. However, because of their closed-access regime, this high cultural ESs capacity does not translate into flow in the valli da pesca dedicated to aquaculture and hunting (groups F and H). Such comparison sheds light on a trade-off between the willingness to maintain high provisioning ESs capacity and flow and the subsequent necessity to lower the flow for cultural ESs to avoid disturbance due to widespread human presence.

In contrast, the valli da pesca that are not managed are broadly frequented by visitors and register a considerable flow, quite as much as the valli da pesca that are purposely managed to offer recreational opportunities.

The difficulty in maintaining high capacities in all three categories of ESs in the attempt to maximize multiple ESs was evident in the M group. Indeed, when considering the relationship between capacity and flow, it turns out that some of the valli da pesca belonging to group M had a flow that exceeded the capacity of provisioning ESs. Moreover, in group M the difference between capacity and flow was lower than in the valli da pesca that focus on one category of ESs.

This result may be related to the necessity to subdivide interventions to maintain the capacity for different categories of ESs simultaneously, resulting in less effective maximization of provisioning ESs capacity.

Because of this contrasting relationship, a negative correlation was also found between the capacity of provisioning ESs and the flow of cultural ESs, explained by the fact that the maximization of provisioning ESs hampers tourists’ access and vice versa. The negative correlation also suggests that building structures and consolidating land to pander for tourist enjoyment can affect the interest in maintaining the characteristics needed to ensure the capacity for provisioning ESs, namely aquaculture, hunting, and harvesting of edible plants and honey. Consequently, when a valle da pesca is managed to maximize recreational activities, regulating services are lost along with the important landscape elements that allow the imitation of natural lagoon functioning. This dynamic probably arises because only the sight of a few natural elements are sufficient to enhance the attractiveness and psycho-physical restorativeness^56–59^. Nevertheless, the necessity to ensure a satisfactory flow of paying visitors has pushed managers to add artificial elements, such as piers, buildings, visitors’ centers, and pools, within a landscape arrangement that does not care much about the actual ecological role of saltmarshes and landscape gradients. This also ignores the necessity of ensuring connectivity between the valli da pesca and the lagoon.

This disconnection from the requirement to maintain ecological processes is reflected in the landscape indicators in other groups. On the one hand, the lack of management in group N has led to a preponderance of saltwater over freshwater pools and saltmarshes, which are instead preserved in the managed valli da pesca (groups F, M, H, R). On the other hand, among the valli da pesca still under management, the most detrimental anthropogenic effect on aquatic habitats seems to be related to the neglect of provisioning ESs to maximize cultural ESs.

The correlation analyses confirmed a relationship between the capacity and flow of the same ESs category and between capacities and flows of different ESs categories, likely due to anthropogenic interventions on the landscape with their effects on the ecosystem structure and functioning.

This result suggests that in managed aquatic ecosystems, such as those considered in this study, a concordance between ESs and ecosystem characteristics occurs. Their relationship can be controlled through anthropogenic modifications, as argued by other authors, both in aquatic^60–64^ and terrestrial ecosystems^65^.

Indeed, to maintain adequate provision of ESs over time, managers must ensure that they do not exploit a flow that is too high compared to the capacity. In addition, they must be aware not to put at risk the capacity of a category of ESs because of the implementation of interventions that maximize the supply of another specific category of ESs. Overlooking the links and feedback loops between different landscape features, and between different ESs, can prove harmful to the valli da pesca system. In contrast, understanding them can represent an impactful outcome, especially when attempting to infer the role of anthropogenic interventions in coupling productive activities and conservation practices.

The choice to lower or even abandon provisioning ESs in the artificially managed ecosystems of the valli da pesca could even prove counterproductive, as it could push regulating ESs in the same direction, according to our results. This can be an unexpected outcome because it is often argued that provisioning ESs should provoke detrimental trade-offs with regulating and maintenance services^66,67^. In addition, other authors have reported that implementing other systems to enhance aquaculture ES capacity often leads to environmental issues, such as deforestation and water eutrophication, as well as competition for space^21,28,68,69^. However, this study shed new light on the relationship between regulating and provisioning ESs in artificially managed ecosystems, fostering a new perspective on the side effects of anthropogenic management through empirical nature-based solutions aimed to enhance ESs capacity.

According to this perspective, the need to grant a high capacity for provisioning ESs in the valli da pesca through anthropogenic interventions boosts the conservation of some habitats and processes.

As an example, the valli da pesca devoted to aquaculture are managed to ensure the persistence of both the presence of fingerlings and salinity gradients. In contrast, the valli da pesca that maximize hunting implement a series of landscape modifications to maintain suitable wintering and nesting habitats, despite the main water body of the lagoon having lost several of such natural characteristics^34,36,37,70^. Besides enhancing provisioning ESs, those efforts can also maintain the presence of some species and support their lifecycles in the long run, confirming the observation of other authors^34,50^.

Consequently, the strategy of implementing measures to imitate the natural processes of lagoon ecosystems to ensure a high provisioning ESs capacity brings up also other ESs, belonging to both regulating and maintenance and cultural ESs categories. Revenues from provisioning ESs flow, in turn, allow managers to implement nature-based solutions and anthropogenic interventions again to sustain the high ESs capacity on which they rely, as if the management was a continuous attempt to conserve and restore the original functioning of the natural ecosystem. In contrast, other management strategies that neglect aquaculture and hunting to focus on enhancement for recreational activities tend to forget the conservation of some critical functions of these areas and, in the long run, might be detrimental to ESs relevant for the entire lagoon.

Therefore, a broad set of ESs may be sought in the valli da pesca, probably including more than the nine evaluated in this study. However, to harness these ecosystems, maximizing the provisioning ESs for which they were born is preferable. Indeed, as long as extensive aquaculture and hunting are considered important, the valle da pesca is arranged to maintain the peculiarities of a transitional water ecosystem’s landscape and ecological processes. In contrast, when a new kind of profitable management distorts the necessity for such high maintenance, the valli da pesca not only lose the capacity and flow of provisioning ESs, but also lose the landscape characteristics on which the regulating ESs rely, resulting in a lower contribution to the entire lagoon ESs.

In light of these considerations, we must consider that all the challenging and unceasing anthropic interventions aimed at conserving the best condition for the valli da pesca require high intellectual and financial effort.

This poses a long-standing dilemma within Venice lagoon^71,72^ regarding the legitimacy of the valli da pesca remaining under private management^73^. If these areas are let under public management, we ensure free and fair public access but may risk losing vital lagoon habitats and, consequently, their regulating and provisioning ESs. On the other hand, allowing private people to maintain them for aquaculture and hunting purposes means ensuring the conservation of regulating and maintenance ESs, along with other ESs, but with the trade-off of a restricted access regime and with the economic advantages that benefit only a few people. Nonetheless, as this work suggests, it is probably because the valli da pesca are under private management that they have maintained the natural ecological processes, along with the landscape features, that make them capable of providing regulating and maintenance ESs.

Therefore, a paradox arises considering that the Venice lagoon ecosystem strongly needs human intervention, mainly privately funded, to conserve its most natural environments and important habitats.

Moreover, in the forecast of ruling the Venice lagoon using mobile barriers to face climate change effects^74,75^, great attention should be paid to hydrodynamic changes resulting from MOSE barrier functioning. Since the artificially managed ecosystems of the valli da pesca exchange matter and energy with the main water body of the lagoon, they could be affected by reduced tidal energy and lowered quality of their waterfront^75–79^.

In conclusion, we highlight that not only do the management strategies affect the ESs in the valli da pesca, but also the Venice lagoon governance does. Thus, we suggest that the decision-makers seek an ecosystem-based management approach that considers all parts of the lagoon, including these managed areas, because they provide several ESs to the entire lagoon system and play an important role within the context of the lagoon conservation.

Furthermore, the valli da pesca clearly show the effect of the anthropogenic modifications that have successfully maximized a specific ES while ensuring sustainability and minimizing the trade-offs between exploitation and environment conservation. The knowledge of these study cases^80^ could inspire the future management of the valli da pesca and can be applied to other similar areas that are part of transitional aquatic ecosystems.

## Methods

The valli da pesca of the Venice lagoon deliver several Ecosystem Services (ESs). In this study, we focused on “regulating & maintenance”, “provisioning”, and “cultural” ESs categories, as defined by the CICES framework nomenclature^41^. The analysis was based on the update of the first spatially explicit assessment of 9 Ecosystem Services: climate regulation, water purification, and lifecycle support (regulating and maintenance ESs); aquaculture production, waterfowl hunting, and wild food and honey (provisioning ESs); tourism, information for cognitive development, and birdwatching (cultural ESs). ESs indicators are reported in Table 2.

**Table 2:** Ecosystem services indicators applied in the assessment.

CICES section	Ecosystem Service	Capacity indicator	Flow indicator
Regulating and Maintenance services	Climate regulation	Carbon sequestration rate by saltmarshes and seagrasses (gC m^−2^ y^−1^)	
	Water purification	Percentage of Nitrogen load removed by denitrification process in brackish water (%)	
	Lifecycle support for fish and avian migratory species	Attractiveness for juvenile fish and migratory waterbirds [0–1 scale]	Number of censused migratory waterbirds (n ha^−1^ y^−1^) and sown fry biomass (kg ha^-1^ y^−1^)
Provisioning services	Aquaculture production	Fish biomass (kg ha^−1^ y^−1^)	Fish biomass (kg ha^−1^y^−1^)
	Waterbirds’ hunting	Number of huntable waterbirds (n ha^−1^ y^−1^)	Huntable waterfowl catch (n ha^−^ y^−1^)
	Wild food	Harvestable *Salicornia sp.* biomass (kg ha^−1^ y^−1^) and honey (kg ha^−1^ y^−1^)	Harvested *Salicornia sp.* biomass (kg ha^−1 ^y^−1^) and honey (kg ha^−1^ y^−1^)
Cultural services	Tourism	Tourism attractiveness [0–1 scale]	Number of tourists (n y^−1^)
	Information for cognitive evelopment	Environmental education attractiveness [0–1 scale]	Number of one-day guided excursionists and students (n y^−1^])
	Birdwatching	Birdwatching attractiveness [0–1 scale]	Mean number of active birdwatchers (n y^−1^)

The ESs assessment was conducted quantifying both “capacity” and “flow”, to evaluate simultaneously the capability of these artificial ecosystems to provide ESs andhe actual amount of ESs they provide to the society^42^.

The spatially explicit evaluation of the capacity and flow of ESs required collecting and processing data from the literature, official harvest and sales records, and quantitative information on the presence of visitors, according to the methods already implemented by Rova et al.^81^ and Stocco et al.^40^, as detailed in the following paragraphs.

### Ecosystem Services assessment methods

#### Regulating and maintenance ESs

The estimation of sequestered carbon in the valli da pesca was considered a proxy for the climate regulation ES. We referred to the annual carbon sequestration by common reed stands^82,83^, vegetated saltmarshes accretion^84,85^, and seagrass meadows^86–89^.

The geographical location of these elements was first determined in 2019 through field surveys in 6 valli da pesca (within areas n. 1, n. 5, n. 10, n. 11, n. 20, n. 21). Then, using regression models, we identified the predictive reflectance values in the R, G, B bands to infer the filtering threshold for the aerial photograms granted by Regione Veneto. The resulting raster maps, with 25 × 25 m resolution, allowed for the detection of saltmarshes, reed stands, and seagrass meadows in the other valli da pesca, where a field survey was not achievable. Each patch extension in m^2^ was associated with its carbon sequestration value, according to the literature ^82,83^. For seagrass meadows, the additional contribution to carbon immobilization due to carbon deposition from microcalcareous epiphytic seaweeds^90^ was considered.

According to the literature, the capacity and flow indicators of climate regulation ESs are considered equivalent because they occur at the same location^91,92^.

To assess the water purification ES, we considered nitrogen removal potential^67,93,94^. We evaluated nitrogen removal in the brackish water basins of the valli da pesca, based on the chemical monitoring of Italian valli da pesca reported by Ravagnan^32^. The capacity and flow were considered equivalent according to the literature^91,92^.

To assess lifecycle support capacity, we focused on the migration patterns of both fish and waterbirds because of the well-known role of the valli da pesca in fish and waterbird migration^34–37,95–97^. The capacity indicator is expressed on a 0–1 normalized scale, where 0 stands for “no potential support” and 1 stands for “maximum potential support”.

To evaluate the support for fish lifecycle, areas with suitable characteristics for effective fish migration were detected based on the result of an Ecosim-Ecopath with Ecospace food-web model, recently spatialized for the Venice lagoon^98^.

To evaluate the support for migratory waterbirds, we mapped the factors that foster the attractiveness of resting and molt changes by adding the positive contribution of saltmarshes, freshwater, shrubs, and herbaceous vegetation^99–101^. Both support contributions were aggregated with ma*p-*algebra operations and normalized to a 0–1 scale.

The lifecycle support ES flow for fish refers to the actual fish biomass sown per hectare (kg/ha/y), as declared by each manager, who decides which species and how much fish to seed based on the potential amount of biomass sustained by the valle da pesca, the availability of fry, and economic factors. The lifecycle support ES flow for waterbird was estimated by calculating the average number of migratory waterbirds that regularly winter within the valli da pesca, calculated from the waterbird annual censuses performed from 2010 to 2020^97^. Resulting lifecycle support flow indicators were normalized to a 0–1 scale and summed to represent an aggregated indicator.

#### Provisioning ecosystem services

The aquaculture ES capacity was estimated as the potential biomass density that can be grown (kg/ha/y) according to the food web model of the Venice lagoon^98^. The flow was expressed as average fish catches per hectare of brackish water surfaces per year (kg/ha/y), according to 2010–2019 official data (granted by Regione Veneto).

The capacity of waterfowl hunting ES was evaluated from the geospatial interpolation of the presence of huntable waterfowl, extracted from the annual waterbird censuses from 2010 to 2020^97^. The hunting flow is the actual waterbird catch per hectare per year, as recorded in the hunting registers of each valle da pesca from 2010 to 2020.

Wild food ES takes into consideration the edible wild plants of the genus Salicornia and the honey that can be obtained from the flowers of sea lavender (a halophytic plant of the genus Limonium).

To locate halophytic vegetation patches, we filtered a series of Worldview-2A satellite images of the study area through a range of annual NDVI averages. The goodness of fit of the detection algorithm was assessed by comparison with vegetational patches, geolocated and analyzed using the visual census method in two different valli da pesca (areas 1 and 8). The spatialized patches were associated with the expected kilograms of Salicornia biomass that could potentially be harvested in the valli da pesca per year^102^. Potential honey production was estimated by considering grams of honey potentially produced per unit area covered by sea lavender flowers^103^.

Wild food ES flow refers to the kilograms of harvested plants and honey: edible plants harvesting data were obtained by local market data and interviews to 14 food & beverage companies, while the amount of the produced sea-lavender honey was quantified through interviews to 6 beekeepers.

#### Cultural ecosystem services

The tourism ES capacity is expressed in terms tourists’ attractiveness. To evaluate it with the spatially explicit approach, we mapped the attractiveness factors witnessed by tourists during a survey conducted in 2019. The most important elements that were ranked were saltmarshes presence, fauna occurrence, good water quality, and visual proximity of natural terrestrial habitats. Each element was weighted to depict the interests declared by the people and normalized to a 0–1 scale index.

Tourism flow is the number of people who, during a year, have spent at least one night in one of the accommodation facilities within the valli da pesca.

The assessment of the capacity for information for cognitive development ES made use of the natural factors that enhance touristic attractiveness, mapped along with inclusivity for people with disabilities. The resulting map was normalized to a 0–1 scale. The flow indicator is the number of persons that attend outdoor educational activities or guided one-day trips annually, as reported by 2 touristic guides and 4 associations (ATN Laguna Sud, Cooperativa Limosa, Ente di promozione turistica di Cavallino Treporti, Oasi WWF Valle Averto).

Birdwatching ES capacity was expressed as birdwatching attractiveness evaluated by 30 interviewed birdwatchers. Spatialization was obtained by mapping the factors that are considered important by birdwatchers, namely the presence of pedestrian paths, saltmarshes presence, nesting areas within the visual field, and the probability of observing birds. The birdwatching flow indicator is the mean number of active birdwatchers retrieved from the 2010–2020 activity trends recorded in the Italian birdwatchers’ database^104^.

#### Identification of management groups and classification

The manager of each valle da pesca manages it as his (or her) own enterprise and makes decisions about rules, business, and maintenance interventions to be implemented in the valle da pesca^73^. Hence, managers were the main source of information and data about aquaculture, hunting, and touristic activities carried out in the privately managed valli da pesca, which were retrieved through 54 interviews. During the interviews, we collected information about the principal ES on which the management relied, the periodic anthropogenic interventions, and the rules regarding access in the valle da pesca. Data on fish seeding, fish production, hunting catches, herbs and honey harvesting, and tourist and excursionists hosted per year in the valle da pesca were also collected.

For data regarding the valli da pesca that are no longer managed, we reviewed the available literature^31,105^ and asked the Veneto Region, local police, and ecotourism guides through 12 interviews.

#### ESs aggregated indicators and landscape indicators

The obtained ESs indicators were normalized through min–max value-based normalization^106,107^ on a 0–1 scale, to allow algebraic operations within each category. We then calculated the aggregated capacity indicators as a sum of the normalized capacity indicators and the aggregated flow as the sum of normalized flow indicators within each ESs category.

To explore whether a relationship exists between landscape and ESs delivery, a highly detailed land cover map was created to identify the landscape arrangement. To obtain this, we applied the European Space Agency restricted access data program asking for very high-resolution satellite scenes, collected by Worldview-02, Worldview-03, and GeoEye-01 satellite constellation. A scalable-boosting decision tree algorithm^108^ was applied to classify the multi-spectral images, considering 4 land cover classes: terrestrial land, saltmarshes, brackish water, and freshwater.

The area covered by the classes in each valle da pesca was retrieved through map algebra analyses. Then we calculated several landscape indicators, as reported in Table 3, to mathematically describe the landscape features that characterize the valli da pesca. As ratios, the indicators are dimensionless quantities, except for the saltmarshes perimeter/total area ratio, which is expressed in km/km^2^.

**Table 3:** Landscape indicators adopted in the study.

Landscape feature	Indicator
Water covered surface extension to the total area	Water area/total area
Terrestrial land coverage	Land area/total area
Saltmarshes coverage	Saltmarshes area/total area
Proportion between freshwater lakes surface and brackish water lakes surface	Freshwater area/brackish water area
Proportion between terrestrial land and saltmarshes area	Land area/saltmarshes area
Saltmarshes perimeter per unit area	Saltmarshes perimeter/saltmarshes area (km/km^2^)
Landscape heterogeneity metric	Landscape shannon’s diversity index
Landscape evenness metric	Landscape shannon’s evenness index

We tested our data for normality using the Shapiro–Wilk test and for homoscedasticity using Bartlett’s test. Because the data were distributed differently from a normal distribution, we chose to perform the Kruskal–Wallis H test on ranks to explore whether at least one group was stochastically dominated by other groups^109^. For significant Kruskal–Wallis test results (p < 0.05), pairwise multiple comparisons among group means were performed using the post-hoc Dunn test^110,111^. This pointed out the significant pairwise difference in the delivery of ESs categories between different groups of valli da pesca.^112^.

The same statistical workflow was used to assess the differences between the groups in terms of landscape indicators.

A unique exception to this statistical approach was represented by the cultural ESs capacity indicator, in which data approximated a normal distribution according to the Shapiro–Wilk test; in this case, an ANOVA test with post-hoc exploratory Tukey pairwise test was preferred.

The analyses were performed using the *raster*^113^ and *fmsb*^114^ packages for the open-source software R 4.1.2^115^ in RStudio 2021.09.2 environment^116^. Land cover classification and spatial analyses were performed using R^115^ integrated with QGIS 3.16.14 Hannover^117^. To test for correlation between capacity and flow in the same ESs category, and between different categories, we calculated Spearman’s rank correlations between ESs indicators to identify mathematical relationship supporting the hypothesis of finding a link between ESs and landscape arrangements under different artificial management conditions.

## Supplementary Information

Supplementary Information.

## Data Availability

The datasets generated and analyzed in this study are not publicly available because they contain personal and business data and thus require special conditions to be given to third parties. However, these are available from the corresponding author upon reasonable request.
