# Supplementary material for: The paradoxical need for human intervention in the conservation of natural environments in Venice lagoon
Source: Sci Rep. 2023 Apr 26;13:6798. doi: 10.1038/s41598-023-33754-3 (PMC10133328; doi:10.1038/s41598-023-33754-3)
Supplement: Supplementary file 1 — Supplementary Information. [file 41598_2023_33754_MOESM1_ESM.pdf]

Supplementary information

The paradoxical need for human intervention in the conservation of natural environments in Venice lagoon

Alice Stocco[1*], Fabio Pranovi[1]

[1] Ca' Foscari University of Venice, Environmental Sciences, Statistics and Informatics Dept., Venezia Mestre, 30174, Italy

* Corresponding author: alice.stocco@unive.it

Supplementary information S1 - Ecosystem Services stellar charts

01 (F)

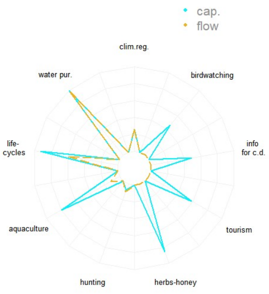

02 (F)

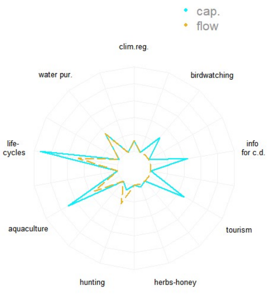

13 (F)

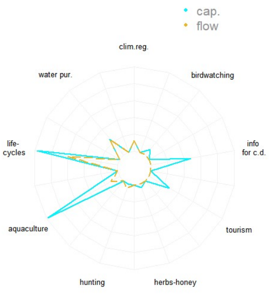

21 (F)

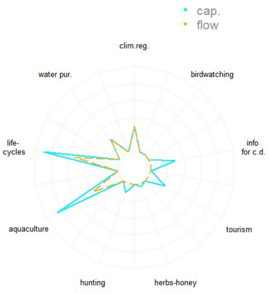

05 (M)

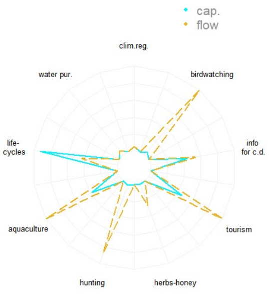

07 (M)

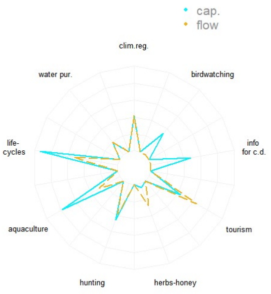

08 (M)

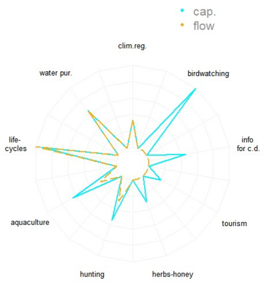

18 (M)

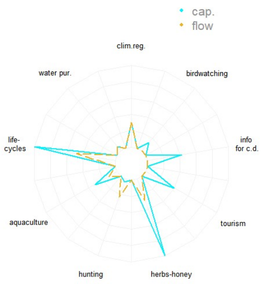

22 (M)

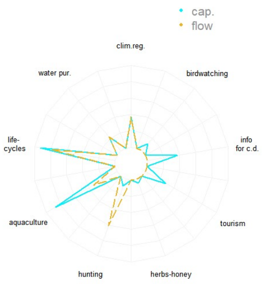

23 (M)

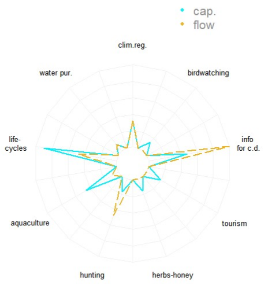

25 (M)

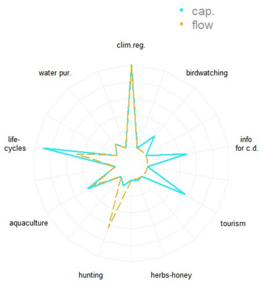

26 (M)

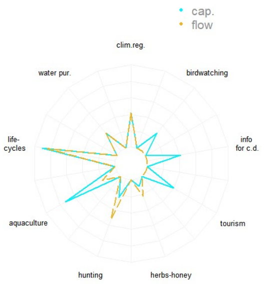

03 (H)

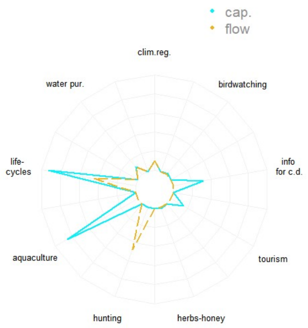

04 (H)

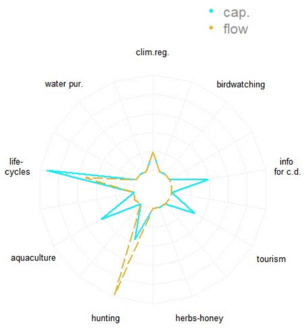

06 (H)

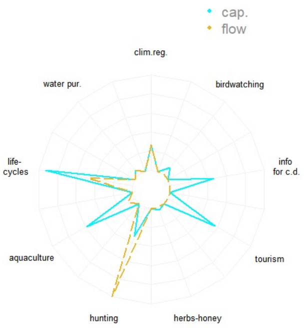

10 (H)

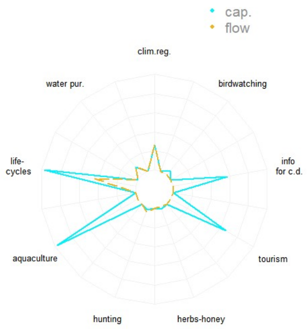

11 (H)

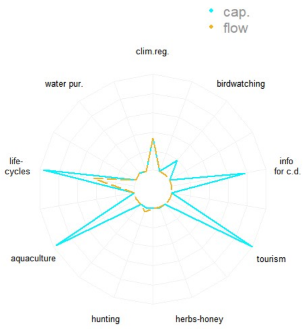

16 (H)

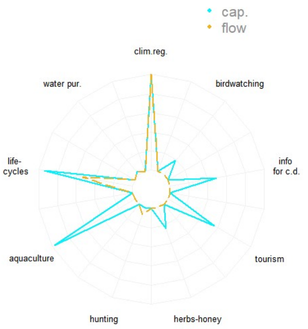

19 (H)

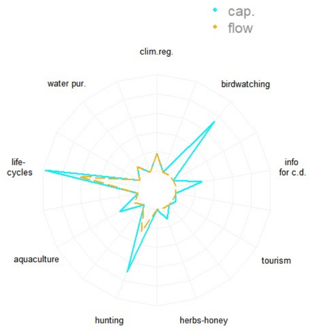

24 (H)

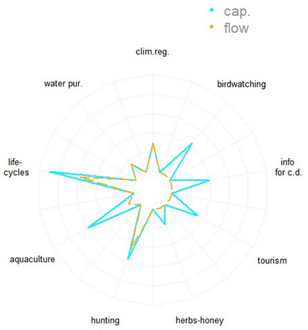

27 (H)

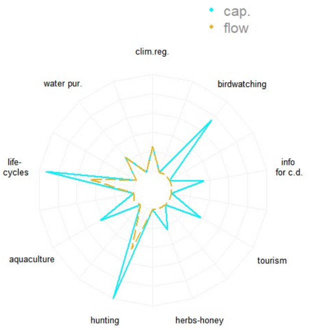

09 (R)

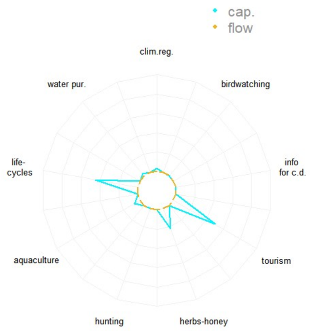

12 (R)

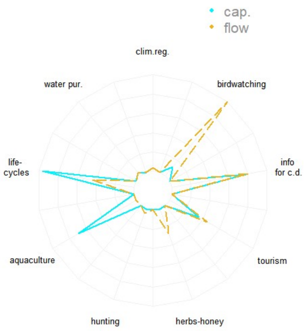

14-15 (R)

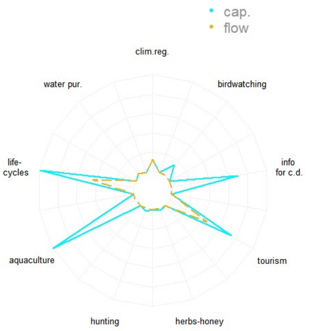

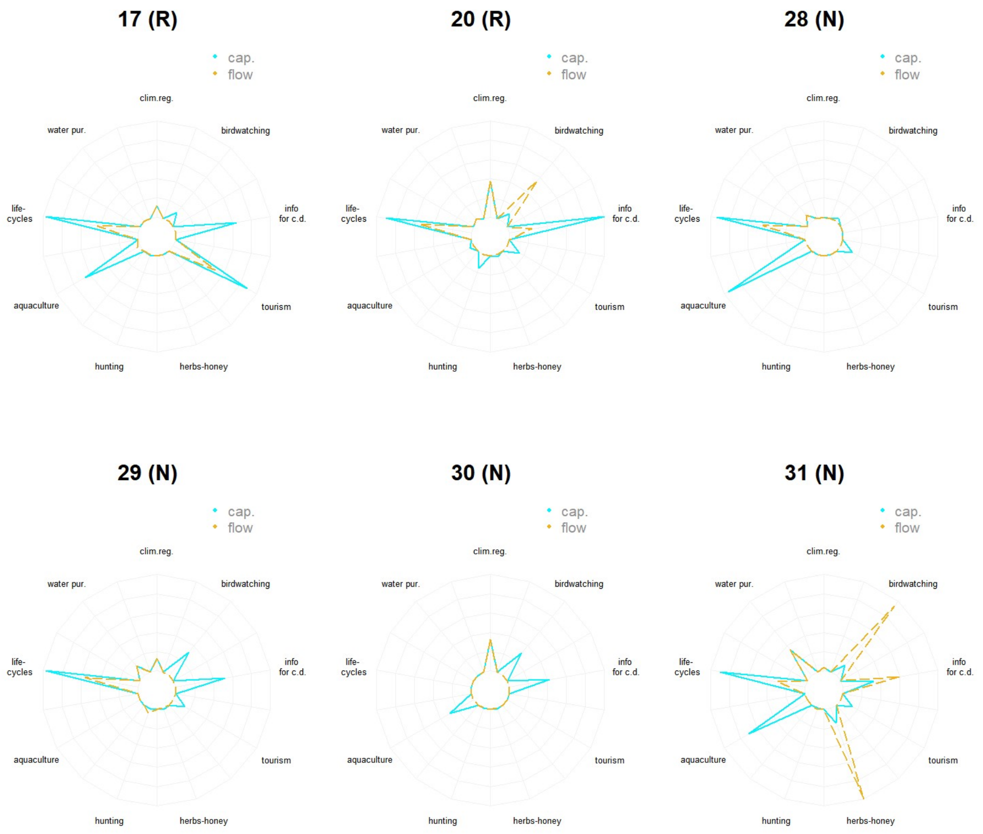

Figure S1- 1 Stellar charts of the Ecosystem Services provided by the valli da pesca. Each plot represents one valle da pesca of the Venice lagoon. The letter in brackets stands for the applied management strategy: F = fish production; M = Multiple ESs; H = Hunting; R = Recreational; N = Not managed. The continuous turquoise lines indicate the capacity to provide the Ecosystem Services labeled in each axis; the dark yellow dashed lines indicate the Ecosystem Services flow.

Supplementary information S2 – Landscape features examples

This section illustrates the landscape features that characterize the different groups of valli da pesca considered in this work. Maps obtained in QGIS 3.16 (https://www.qgis.org) with basemap provided by the author's merging of aerial photographs WMS layers (https://idt2.regione.veneto.it/gwc/service/wmts) with additional processing.

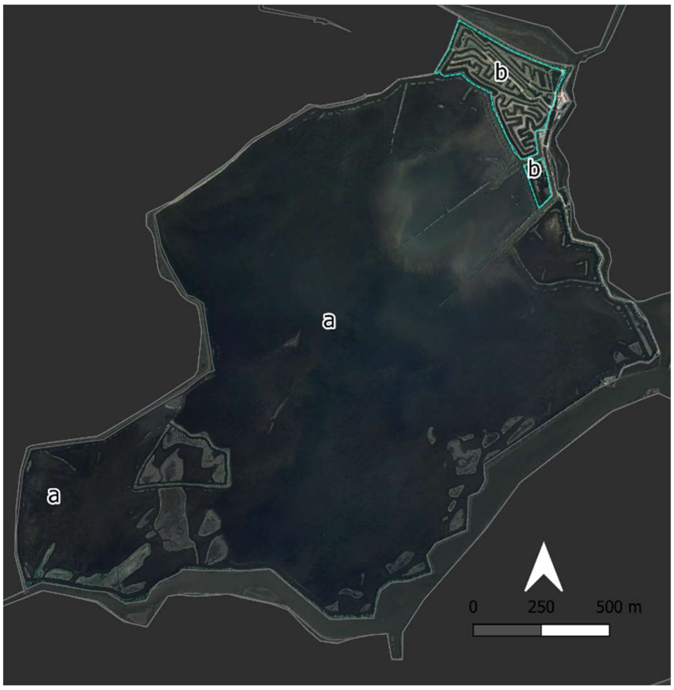

Figure S2.1 A valle da pesca of group F. A large brackish-water basin (a) and the presence of geometrical, deeper fishponds (b) characterize the valli belonging to this group, managed for maximization of extensive aquaculture.

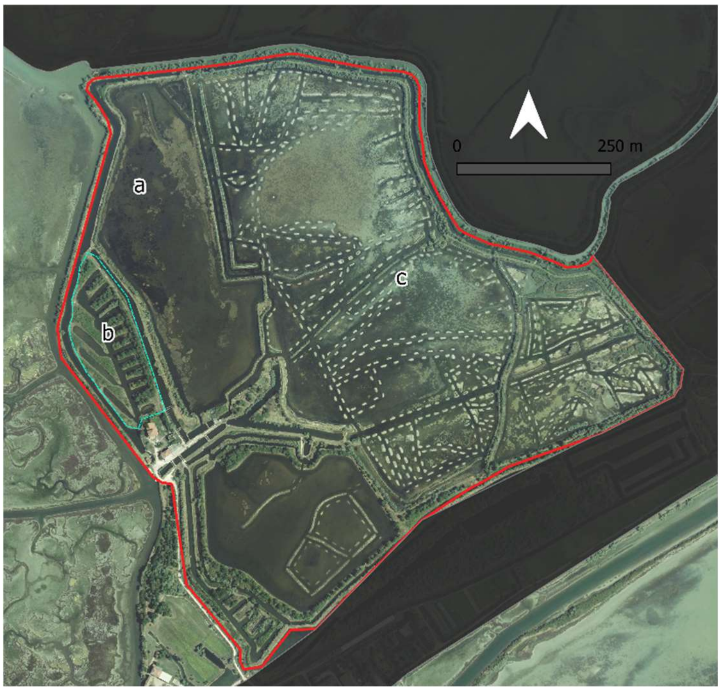

Figure S2.2 A valle da pesca of group H, aiming to maximize hunting ES. Remnants of brackish lakes (a) and wintering fishponds (b), which are no longer used for aquaculture, give way to a series of shallow freshwater lakes (c). Several natural and artificial saltmarshes peculiar to the hunting lakes of these ecosystems are present within the freshwater area (c).

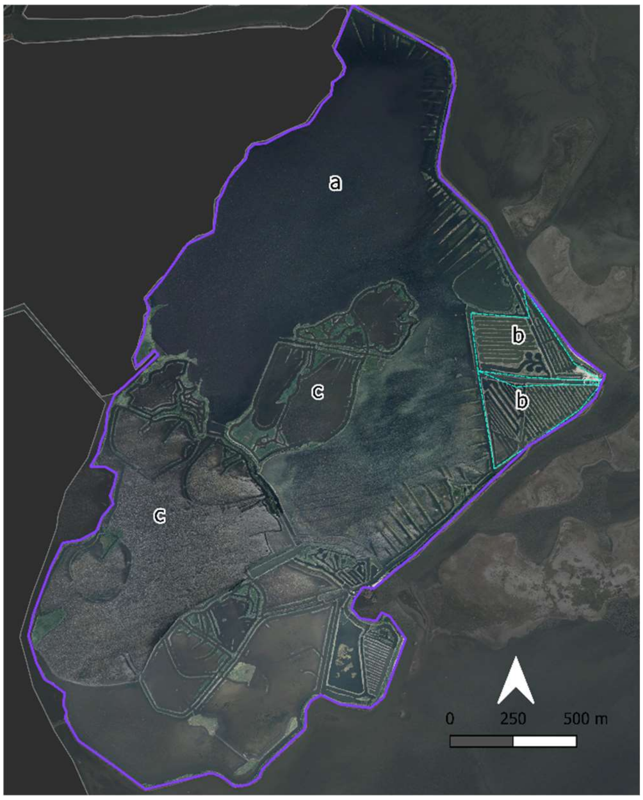

Figure S2.3 A valle da pesca of group M, maximizing aquaculture and hunting. A wide brackish lake (a), the fishponds, and the hunting lakes with vegetated saltmarshes (c) are visible.

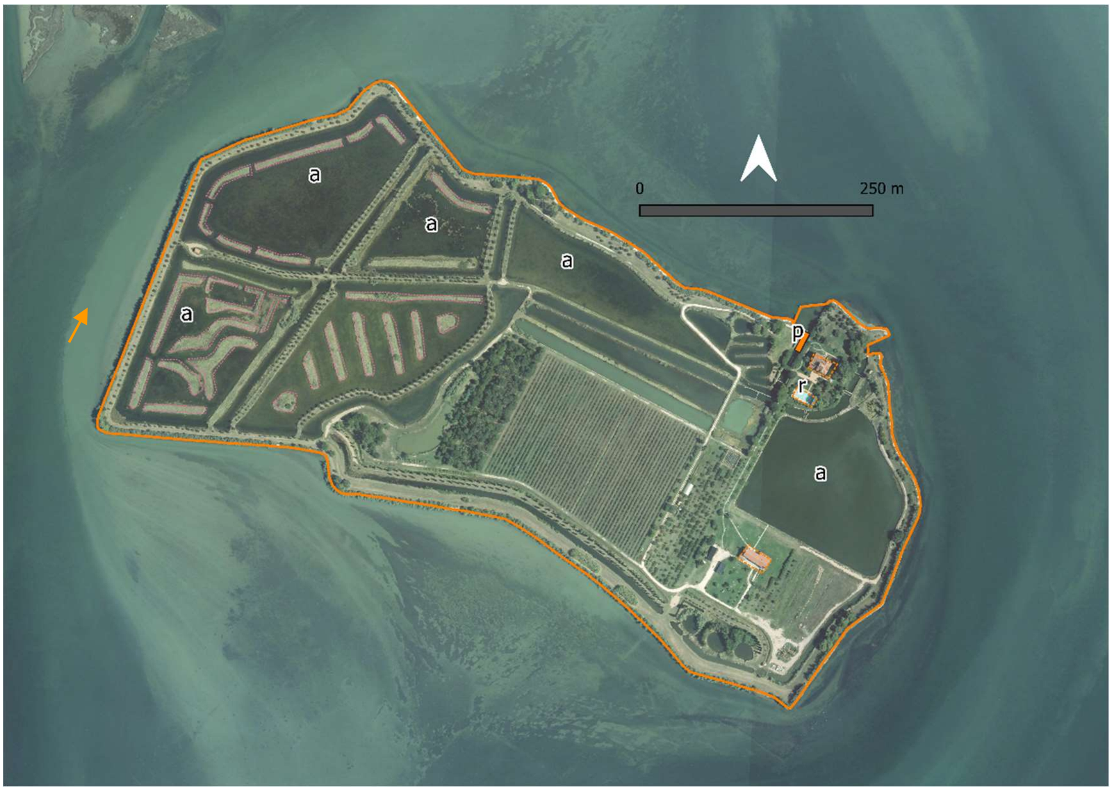

Figure S2.4 A valle da pesca of group R managed to maximize cultural ESs. Terrestrial land covers an area more extended than the area covered by artificial saltmarshes (contoured by dotted pink lines in the image).

Brackish lakes (a) are little if not productive for aquaculture. There are buildings and recreational facilities such as a pool (r), a pier (p), and several walking paths.

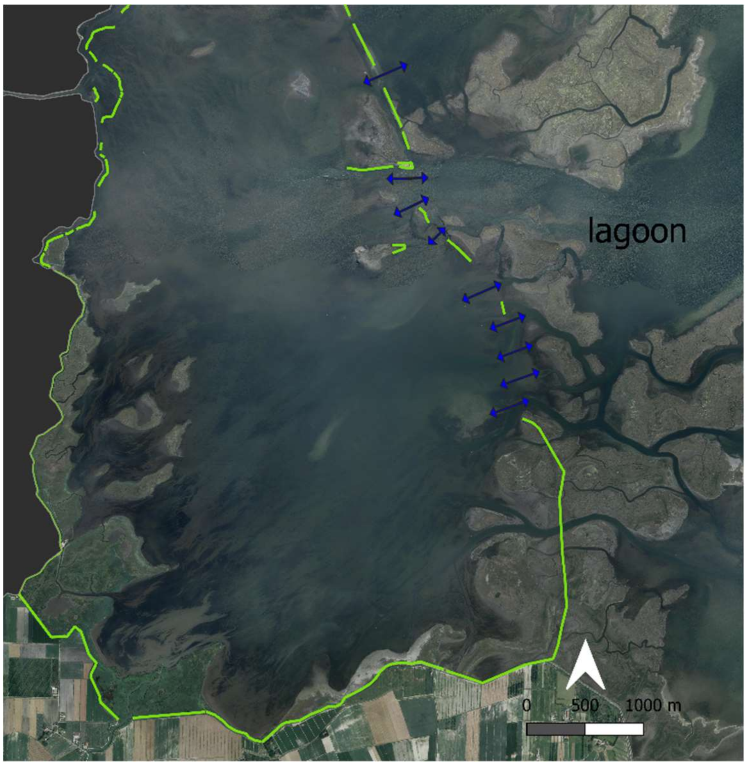

Figure S2.5 A valle da pesca of group N. Only remnants of ancient artificial boundaries and levees are visible (green lines), and saltmarshes are prone to erosion. Connection with the lagoon is total: the water flows freely without human intervention through channels and creeks (blue arrows) between saltmarshes.
